# Methylene Blue Induces Antioxidant Defense and Reparation of Mitochondrial DNA in a Nrf2-Dependent Manner during Cisplatin-Induced Renal Toxicity

**DOI:** 10.3390/ijms24076118

**Published:** 2023-03-24

**Authors:** Natalia A. Samoylova, Artem P. Gureev, Vasily N. Popov

**Affiliations:** 1 Department of Genetics, Cytology and Bioengineering, Voronezh State University, 394018 Voronezh, Russia; nataliya.samoylova.2000@mail.ru (N.A.S.); pvn@bio.vsu.ru (V.N.P.); 2 Laboratory of Metagenomics and Food Biotechnology, Voronezh State University of Engineering Technology, 394036 Voronezh, Russia

**Keywords:** cisplatin, nephrotoxicity, methylene blue, mitochondrial DNA, DNA-reparation, Nrf2/ARE

## Abstract

Cisplatin is a platinum-based cytostatic drug that is widely used for cancer treatment. Mitochondria and mtDNA are important targets for platinum-based cytostatics, which mediates its nephrotoxicity. It is important to develop therapeutic approaches to protect the kidneys from cisplatin during chemotherapy. We showed that the exposure of mitochondria to cisplatin increased the level of lipid peroxidation products in the in vitro experiment. Cisplatin caused strong damage to renal mtDNA, both in the in vivo and in vitro experiments. Cisplatin injections induced oxidative stress by depleting renal antioxidants at the transcriptome level but did not increase the rate of H_2_O_2_ production in isolated mitochondria. Methylene blue, on the contrary, induced mitochondrial H_2_O_2_ production. We supposed that methylene blue-induced H_2_O_2_ production led to activation of the Nrf2/ARE signaling pathway. The consequences of activation of this signaling pathway were manifested in an increase in the expression of some antioxidant genes, which likely caused a decrease in the amount of mtDNA damage. Methylene blue treatment induced an increase in the expression of genes that were involved in the base excision repair (BER) pathway: the main pathway for mtDNA reparation. It is known that the expression of these genes can also be regulated by the Nrf2/ARE signaling pathway. We can assume that the protective effect of methylene blue is related to the activation of Nrf2/ARE signaling pathways, which can activate the expression of genes related to antioxidant defense and mtDNA reparation. Thus, the protection of kidney mitochondria from cisplatin-induced damage using methylene blue can significantly expand its application in medicine.

## 1. Introduction

Cytostatics are common drugs which are widely used for the treatment of cancer [1]. Within chemotherapy, cytotoxic drugs are used as active agents to treat rapidly spreading tumor cells by inhibiting proliferation, inducing apoptosis, damaging DNA, or disrupting the cell metabolism [2]. Cisplatin is a divalent platinum ammonium chloride complex [3]. It is a widely used chemotherapeutic agent for the treatment of various types of malignant neoplasms, such as melanoma, lymphoma, carcinoma, sarcoma, and germ cell tumors [4], and it has been used for many decades with a significant increase in survival rates [5]. The introduction of cisplatin into therapy has completely changed the prognosis of some cancers. Many studies now state that between 50 and 70% of chemotherapy patients are treated with platinum-based drugs [6]. The anti-tumor effect of the cisplatin is based on its ability to cause the formation of coordination bonds between the two purine bases of DNA and the platinum atom by alkylation. DNA–cisplatin adducts lead to distortions in the structure of the double helix due to the formation of interstrand and intrastrand crosslinks, which disrupt the mechanisms of DNA replication and transcription, delaying the cell cycle and promoting apoptosis [7].

Unfortunately, cytostatics do not have a local effect and impact on the whole organism. The main limitation of the cisplatin treatment is nephrotoxicity, which occurs in about a third of patients, and usually appears about 10 days after treatment [8]. The sensitivity of the kidneys to the cisplatin is likely related to their major role in its excretion [9]. Due to its low molecular weight and uncharged nature, unbound cisplatin is freely filtered by the glomeruli, and most of it is retained in the renal cortex. The kidneys accumulate platinum in part by transport or specific binding to the main transport system and biotransform it intracellularly [10]. In addition, there is an increase in the production of tumor necrosis factor-α (TNF-α) and reactive oxygen species (ROS), which stimulates inflammation, oxidative stress, vascular damage, necrosis, and apoptosis pathways [11]. These events collectively lead to the vasculature damage, reduction of blood flow, ischemic damage, loss of organ function, and acute renal failure [12], recurring episodes of which can lead to chronic kidney disease [13].

Mitochondria, in particular mitochondrial DNA (mtDNA), are also targets for cisplatin [14]. mtDNA is more sensitive to damage, since it lacks some repair mechanisms compared to nuclear DNA [15]. In addition, mtDNA lacks histones and is located in the mitochondrial matrix, which is in close proximity to the main site of ROS production in the cell [16]. Kidney cells have a high mitochondrial density since the reabsorption of glomerular filtrate requires extremely high energy [17]. For this reason, cisplatin-induced mitochondrial dysfunction may be an important link in the chain of processes leading to chronic kidney disease. 

The clinical limitations of the cisplatin motivate researchers to create thousands of its analogs [18], but most of these compounds do not have a significant advantage over cisplatin [19]. For this reason, the search for drugs which may reduce the side effects of cisplatin without reducing its therapeutic effect is an important task for pharmacology. It is preferable to use drugs that have a protective effect against nephrotoxicity. Methylene blue is a heterocyclic compound that appears to be an effective drug. Despite the almost 120-year history of methylene blue in medicine, interest in this compound as a potential protector against cisplatin toxicity has appeared only in the last 2 years. It was shown that methylene blue reduces levels of caspases in tissue, creatinine, and blood urea nitrogen in the kidneys [20], restores sperm function in the testes [21], and reduces mtDNA damage in the brain [22] after treatment with cisplatin. The effect of methylene blue on kidney mtDNA damage has not been studied previously. We hypothesize that methylene blue may have a nephroprotective effect by the same mechanism as the neuroprotective effect.

The goal of this study was to evaluate the effect of cisplatin, methylene blue, and its metabolite azure B on the levels of ROS production, lipid peroxidation, and mtDNA damage in isolated kidney mitochondria, establish the protective effect of methylene blue and azure B under cisplatin-induced renal toxicity in vivo, and evaluate their effect on the expression levels of the main cytoprotective genes.

## 2. Results

### 2.1. Effect of Cisplatin and Thiazine Dyes on the Rate of H_2_O_2_ Production in Kidney Mitochondria

**Figure 1:**
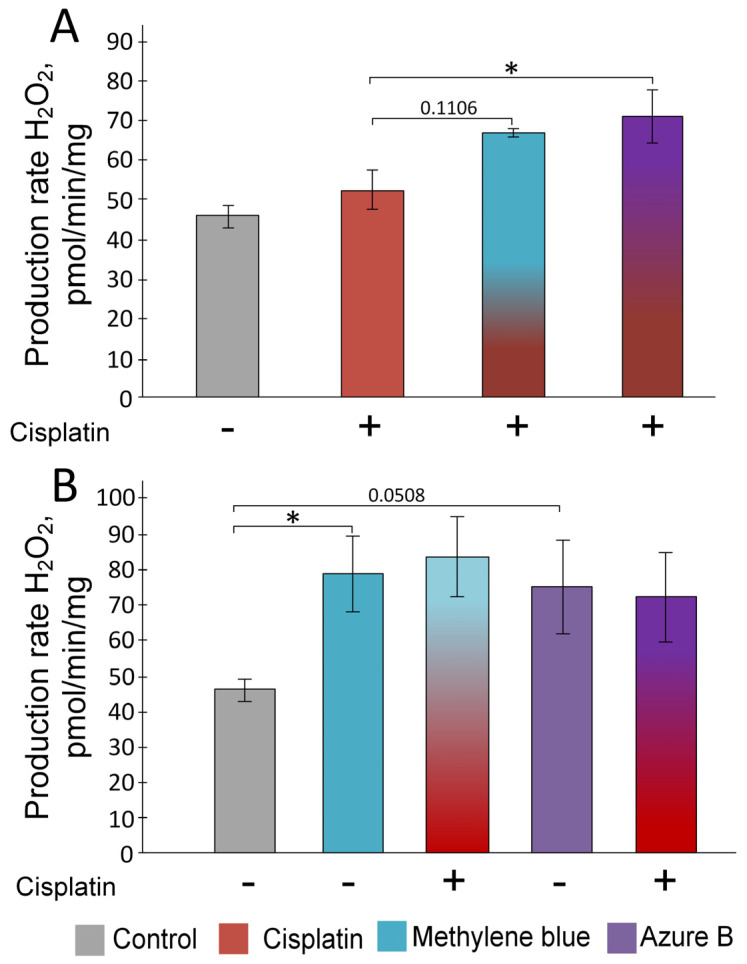
Impact of cisplatin, methylene blue, and azure B on the rate of H_2_O_2_ production: (**A**) Addition of cisplatin did not increase the rate of H_2_O_2_ production, and subsequent additions of methylene blue or azure B increased it; (**B**) Addition of methylene blue or azure B increased the rate of H_2_O_2_ production, and subsequent additions of cisplatin did not impact on the rate of H_2_O_2_ production. The results are expressed as means ± SEM. * Differences are statistically significant *p* < 0.05 (Mann–Whitney test). All measurements were provided in at least four repetitions.

The addition of cisplatin did not significantly increase the rate of H_2_O_2_ production in intact mouse kidney mitochondria (Figure 1). There was a change in the rate of H_2_O_2_ production from 46.06 ± 0.27 pmol/min/mg to 52.54 ± 5.21 pmol/min/mg. The addition of methylene blue increased the rate of H_2_O_2_ production to 66.81 ± 0.97 pmol/min/mg, but the differences were not statistically significant (*p* = 0.061). The addition of azure B increased the rate of H_2_O_2_ production to 70.97 ± 6.85 pmol/min/mg (*p* < 0.05).

Preliminary addition of methylene blue to isolated mitochondria caused more significant increases in the rate of H_2_O_2_ production (to 83.68 ± 11.42 pmol/min/mg protein, *p* < 0.05), as well as the addition of azure B (to 75.12 ± 13.52 pmol/min/mg protein, *p* = 0.05). Subsequent addition of cisplatin did not cause any change in the rate of H_2_O_2_ production. 

### 2.2. The Effect of Cisplatin and Thiazine Dyes on the Levels of Lipid Peroxidation Products in the Kidneys

**Figure 2:**
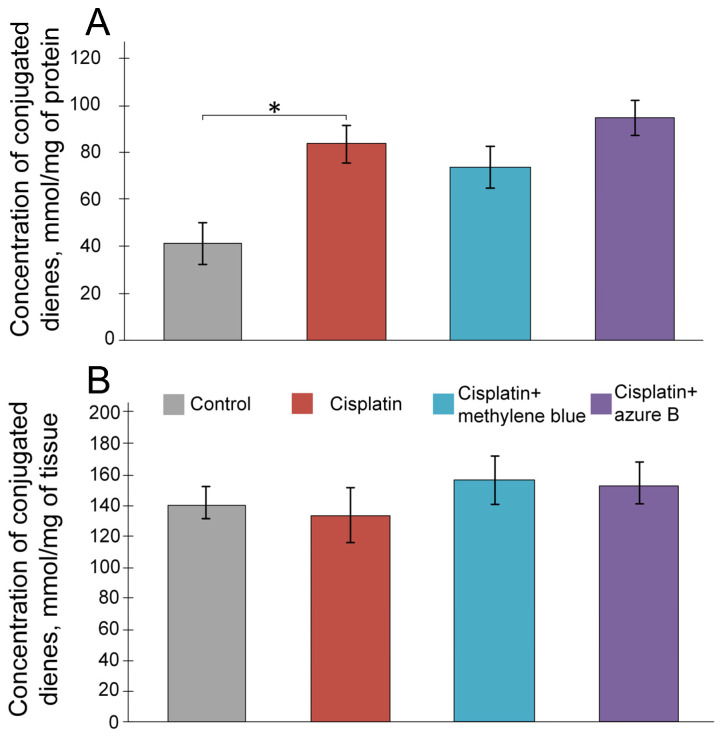
(**A**) Concentration of conjugated dienes in the in vitro experiment. Adding cisplatin to mitochondria increased the concentration of conjugated dienes. Subsequent additions of methylene blue or azure B had no effect on the concentration of the diene conjugates. The results are expressed as means ± SEM. All measurements were provided in at least four repetitions. (**B**) Concentration of conjugated dienes in the in vivo experiment. Cisplatin injection and methylene blue and azure B treatment did not impact on the concentration of conjugated dienes. * Differences are statistically significant *p* < 0.05 (Mann–Whitney test). Control (*n* = 10), cisplatin (*n* = 6), cisplatin + methylene blue (*n* = 8), cisplatin + azure B (*n* = 8).

During an in vitro experiment, we showed a two-fold increase in the concentration of DC in mitochondria incubated with cisplatin compared with the control (*p* < 0.05). Preliminary incubation of mitochondria with solutions of methylene blue and azure B did not affect the cisplatin-induced increase in DC concentration (Figure 2A). During an in vivo experiment, we did not show statistically significant differences between the studied groups (Figure 2B).

### 2.3. Effect of In Vitro Cisplatin and Thiazine Dye Addition to Mitochondria on mtDNA Damage Levels

**Figure 3:**
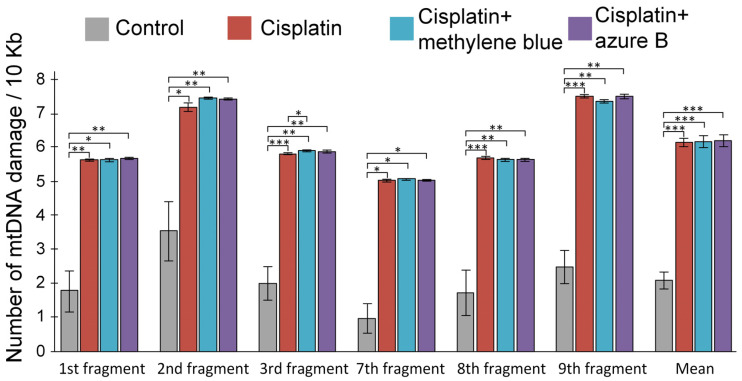
Impact of cisplatin, methylene blue, and azure B addition to intact renal mitochondria on the number of mtDNA damage. Differences are statistically significant: * *p* < 0.05, ** *p* < 0.01, *** *p* < 0.001 (Mann–Whitney test). The results are expressed as means ± SEM. All measurements were provided in at least four repetitions.

During an in vitro experiment, we showed that cisplatin caused strong damage in all studied fragments of mtDNA (Figure 3). A five-fold increase in the amount of mtDNA damage was observed in the 7th fragment (*p* < 0.05). A three-fold increase in the number of mtDNA damage was observed in the 1st fragment (*p* < 0.01), 3rd fragment (*p* < 0.001), 8th fragment (*p* < 0.001), and 9th fragment (*p* < 0.001). In the 2nd fragment, a two-fold increase in the number of mtDNA damage in the cisplatin-treated mitochondria was observed (*p* < 0.05). Preincubation of mitochondria with methylene blue or azure B did not prevent cisplatin-induced lesions of mtDNA.

### 2.4. Effect of Cisplatin Injections and In Vivo Administration of Thiazine Dyes on Levels of mtDNA Damage

**Figure 4:**
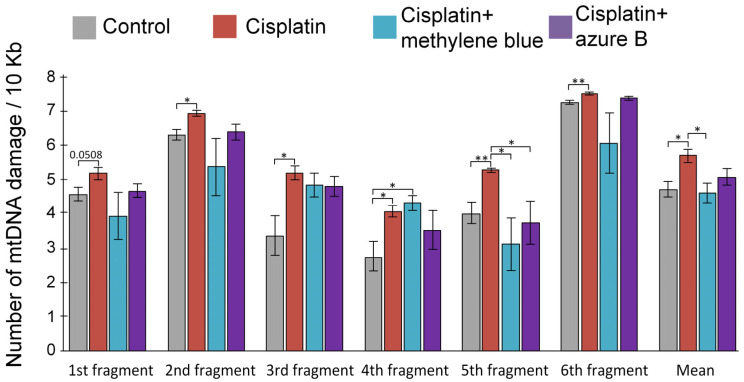
Impact of cisplatin injections and methylene blue and azure B treatments on the amount of mtDNA damage in kidneys. Differences are statistically significant: * *p* < 0.05, ** *p* < 0.01 (Mann–Whitney test). The results are expressed as means ± SEM. Control (*n* = 10), cisplatin (*n* = 6), cisplatin + methylene blue (*n* = 8), cisplatin + azure B (*n* = 8).

An analysis of the average number of mtDNA showed that cisplatin injection increased the amount of damage for 20%, compared with the control (*p* < 0.05) (Figure 4). Methylene blue pre-treatment prevented the cisplatin-induced mtDNA lesion (*p* < 0.05 compared with cisplatin-treated mice). Azure B pre-treatment prevented the cisplatin-induced mtDNA lesion, but statistically significant differences were not observed with cisplatin-treated mice.

In the 1st, 2nd, 3rd, and 9th fragments, cisplatin injections induced an increase in the number of mtDNA damage (*p* = 0.05 for the 1st fragment, *p* < 0.05 for the 2nd and 3rd fragments, *p* < 0.01 for 9th fragment). In these fragments, pre-treatment with methylene blue and azure B prevented the increase in the amount of mtDNA damage; additionally, statistically significant differences were not observed with cisplatin-treated mice. An increase in the amount of mtDNA damage both in the cisplatin and cisplatin + methylene blue groups compared with the control was observed in the 7th fragment (both *p* < 0.05). Methylene blue and azure B statistically significantly decrease the number of mtDNA, compared with the cisplatin-treated mice in the 8th fragment (both *p* < 0.05).

### 2.5. Effect of Cisplatin and Thiazine Dyes on Gene Expression Levels

**Figure 5:**
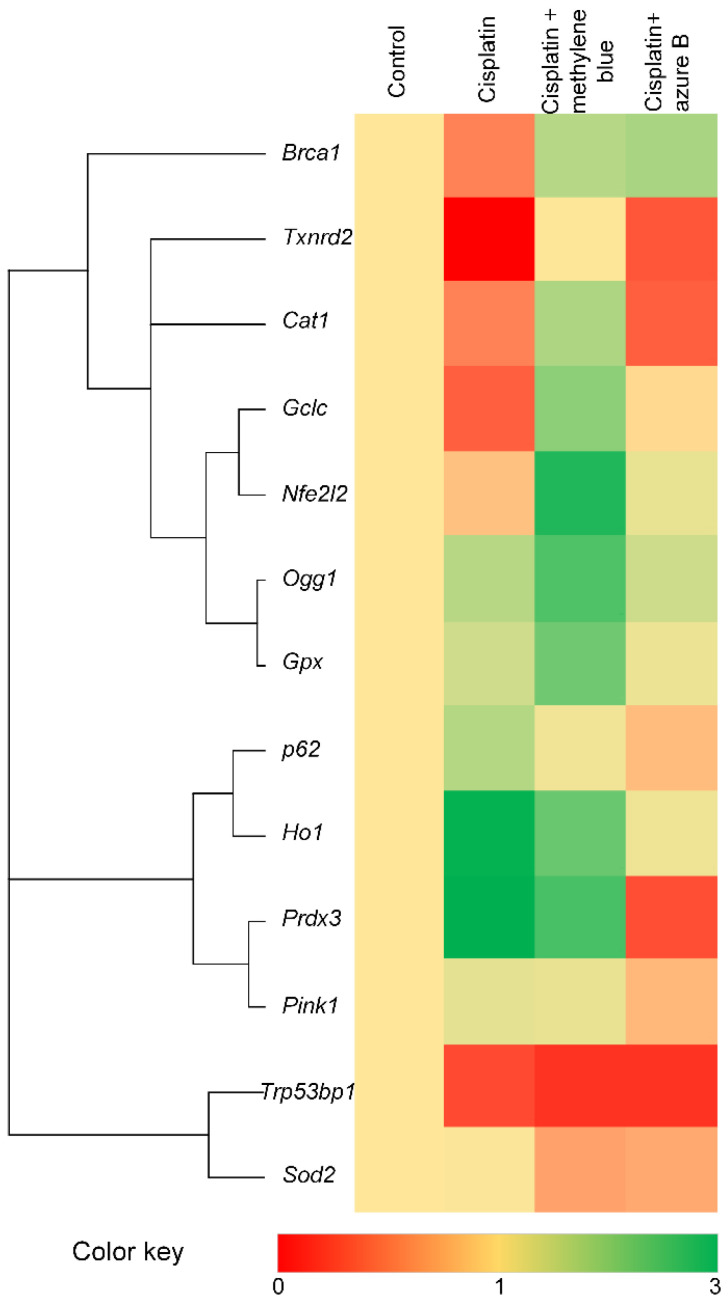
Heat-map of change in the gene expression in the kidneys after cisplatin injections and methylene blue and azure B treatments. Genes are grouped according to their similarity in expression profile. Control (*n* = 10), cisplatin (*n* = 6), cisplatin + methylene blue (*n* = 8), cisplatin + azure B (*n* = 8).

Cluster analysis of gene expression reveals three clusters (Figure 5). The first cluster includes the transcription factor *Nfe2l2*, antioxidant genes (*Txnrd2*, *Cat*, *Gclc*, *Gpx*), and genes involved in DNA repair (*Brca1*, *Ogg1*). In this group, the expression of most of the genes decreased after cisplatin injections. We observed a two-fold decrease in the expression of the *Brca1*, *Ogg1*, and *Gclc* genes, and a 95% decrease in the expression of *Txnrd2*. At the same time, the methylene blue treatment increased the expression of the *Brca1*, *Txnrd2*, *Cat*, *Gclc*, and *Nfe2l2* genes by 2–4 times, and increased the expression of the *Ogg1* and *Gpx* genes by 1.5 times. The azure B significantly increased only the expression of the *Brca1* and *Txnrd2* genes.

The second cluster included genes involved in the regulation of mitophagy (*p62* and *Pink1*), the antioxidant gene *Prdx3*, and the gene-encoding heme oxygenase (*Ho1*). Cisplatin injections increased the expression of these genes, especially *Ho1* and *Prdx3*. Methylene blue does not significantly affect gene expression, unlike azure B, which reduced the expression of these genes, especially *Ho1* and *Prdx3*.

The third cluster includes two genes: the antioxidant gene *Sod2* and the gene involved in DNA repair, *Trp53bp1*. Therapy of mice with thiazine dyes had no significant effect on their expression level, while injections of cisplatin significantly reduced the expression of the *Trp53bp1* gene.

## 3. Discussion

Nephrotoxicity is one of the main limitations in cisplatin therapy, despite the high efficacy in the treatment of cancer. Oxidative stress plays an important role in the induction of toxicity by cisplatin [23]. Oxidative stress can arise directly; for example, by increasing the rate of H_2_O_2_ production in mitochondria, which have at least ten ROS production sites: mono amine oxidases A and B, cytochrome b5 reductase, dihydroorotate dehydrogenase, α-glycerophosphate dehydrogenase, complex I, coenzyme Q, complex III, cytochrome c, cytochrome c oxidase, succinate dehydrogenase, aconitase, α-ketoglutarate dehydrogenase complex, and pyruvate dehydrogenase complex [24]. Oxidative stress can also be formed indirectly; for example, by reducing intracellular concentrations of glutathione [25], by reducing the level of NADPH and SH-groups, and by the reduction of expression or activity of key antioxidant enzymes glutathione peroxidase (GSH–Px), glutathione reductase (GR), and glutathione S-transferase (GST) [26,27,28]. In this study, we have shown that the addition of cisplatin to isolated mitochondria did not lead to an increase in hydrogen H_2_O_2_ (Figure 1). This is not surprising, since cisplatin has not previously been shown to cause inhibition of any respiratory chain complexes. In addition, some inhibitors of the respiratory chain are used in co-chemotherapy with cisplatin to increase the level of oxidative stress to damage tumor cells [29]. However, our data do not contradict the claim that cisplatin causes oxidative stress in the kidneys. We have shown a decrease in the expression of the *Cat*, *Gclc*, and *Txnrd2* genes (Figure 5) encoding the corresponding antioxidant enzymes. *Cat* is a gene encoding the catalase, a key antioxidant enzyme in protecting the body from oxidative stress by converting H_2_O_2_ into water and oxygen [30]. The glutathione is a tripeptide that consists of glutamate, glycine, and cysteine. The first reaction of glutathione synthesis is rate-limiting and is catalyzed by glutamate cysteine ligase (GCL), which consists of two subunits: a heavy or catalytic (GCLC, Mr ∼73,000) and a light or modifier (GCLM, Mr ∼30,000) subunit. As a result, γ-glutamyl-l-cysteine is formed, to which glycine is further attached via glutathione synthase (GSS) [31].

Powerful antioxidants that remove peroxide are peroxiredxins (PRDXs), which are able to eliminate H_2_O_2_, alkyl hydroperoxides, and peroxynitrite. All PRDX enzymes are obligatory dimers and contain a conserved NH2-terminal cysteine–SH residue that reacts with H_2_O_2_ to form cysteine sulfenic acid (cysteine–SOH) with the release of H_2_O. Oxidized cysteine residues, the cleavage of disulfide bonds, and the reduction of peroxiredoxins are carried out by proteins from the thyredoxin (TXN) family. In this case, a disulfide bond is formed between cysteine residues in TNX itself. TNXRD2 transfers an electron from NADPH to active TXN. The reaction begins with a reduction of the selenenylsulfide to the selenolate anion with electrons received from NADPH via FAD. The second electron transfer from a second molecule of NADPH reduces the cysteine. The selenolate anion then attacks the disulfide bonds of TXN and the resulting to regenerate the selenenylsulfide. *Txnrd2* encodes the mitochondrial form of thioredoxin reductase, which is important for scavenging ROS in mitochondria [32]. It has previously been shown that cisplatin can induce depletion of renal antioxidant defense systems, such as GST, glutathione peroxidase, superoxide dismutase, catalase, activities, and a reduced glutathione level [33]. Thus, we can conclude that the inhibition of the antioxidant defense of the kidneys on the transcriptome level may be the cause of cisplatin-induced oxidative stress.

It is well known that free radicals interact with membrane lipids, causing their peroxidation [34]. We showed that the addition of cisplatin to mitochondria promotes an increase in the level of DC in the kidney’s mitochondria in vitro (Figure 2A), which corresponds with the data obtained earlier, where it was shown that the treatment of renal cortical slices with cisplatin in vitro leads to an increase in lipid peroxidation products [35]. At the same time, injections of cisplatin did not lead to an increase in the DC level in the in vivo experiment (Figure 2B). These data are inconsistent with studies that have repeatedly demonstrated an increase in the product of lipid peroxidation levels in the kidneys in vivo under the cisplatin treatment. It is likely that the reason for the discrepancy between the results is the measurement of different kinds of lipid peroxidation markers. Previously, the estimation of the concentration of malonic aldehyde was performed [26,36], which are secondary lipid peroxidation products and are formed as a result of the cleavage of oxidized polyunsaturated fatty acids (PUFAs). Malonic aldehyde is a widely used lipid peroxidation marker, but not specific enough, since malonic aldehyde can be formed by the degradation of non-lipid molecules (proteins, bile pigments, nucleic acids, and carbohydrates). DC are the primary products of lipid peroxidation. During the free-radical oxidation of arachidonic acid, hydrogen cleavage occurs in the δ-position with respect to the double bond, which leads to the displacement of this double bond with the formation of DC [37]. For this reason, the concentration of DC can be considered a more reliable indicator of the concentration of lipid peroxidation products [38]. However, deeper studies of the effect of cisplatin on the lipid peroxidation processes are needed.

**Figure 6:**
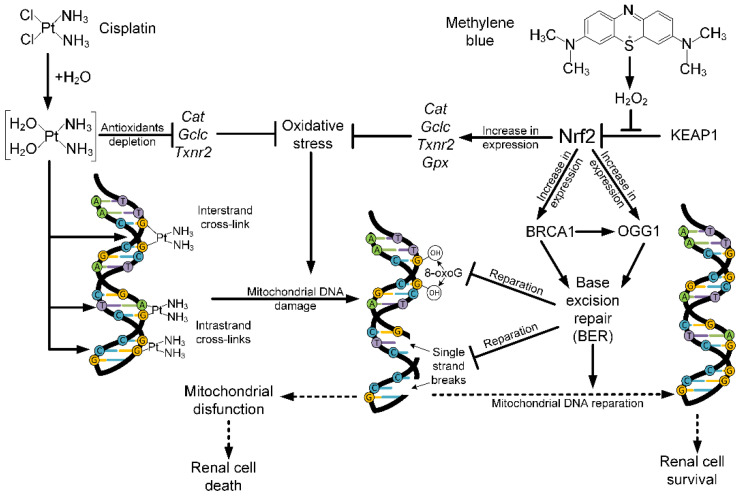
Effect of cisplatin and methylene blue on the oxidative stress and mtDNA damage. Cisplatin penetrates the cell in the aquatic form. Chloride atoms on cisplatin are displaced by water molecules. Cisplatin can cause antioxidant depletion and mtDNA damage by origins of interstrand and intrastrand crosslinks, with subsequent formation of oxidized guanine bases and single-strand breaks. Damage of the mtDNA can cause mitochondrial dysfunction and renal cell death. Methylene blue induces H_2_O_2_ production in mitochondria. H_2_O_2_ can trigger the Nrf2/ARE signaling pathway by oxidation of Keap1. Nrf2 can increase the expression of antioxidant genes for defense against cisplatin-induced oxidative stress. Nrf2 activation leads to increase the expression of genes which are involved in the base excision repair (BER): the main mechanism of mtDNA reparation. BER restores an intact mtDNA structure and protects kidney cells from death.

Oxidative stress not only leads to the damage of biological membranes, but also affects the DNA structure. The damaging effect of cisplatin on nuclear DNA has been well studied [4,39]. Cisplatin causes the formation of coordination bonds between the two purine bases of DNA and the platinum atom by alkylation. The appearance of such adducts of DNA–cisplatin leads to distortions in the structure of the double helix due to the formation of interstrand and intrastrand crosslinks (Figure 6), which disrupts the mechanisms of DNA replication and transcription, as well as induces 8-oxoguanine formation and single-strand breaks, delaying the cell cycle and promoting apoptosis [7].

The mechanism of cisplatin binding to mtDNA is similar to nuclear DNA binding; however, if adducts are removed in nuclear DNA and DNA is restored by nucleotide excision repair, then similar nucleotide excision repair (NER) mechanisms are absent in mitochondria [14]. In both the in vitro and in vivo experiments, cisplatin damaged all studied mtDNA fragments without exception (Figure 3 and Figure 4). According to the data obtained earlier, the treatment of cells with cisplatin leads to the binding of one platinum molecule per 3800 bp of nuclear DNA and the binding of one platinum molecule per 2166 bp of mtDNA [14,40]. This may indicate that cisplatin-induced mtDNA damage is more significant than nuclear DNA damage. MtDNA damage can cause deterioration of energy metabolism in the kidney, and mtDNA mutations are associated with proximal and distal tubular dysfunctions, renal Fanconi syndrome, focal segmental glomerulosclerosis, tubulointerstitial nephritis, etc. [17]. For this reason, mtDNA protection in cisplatin-induced kidney injury is an important task that reduces cisplatin nephrotoxicity.

The various strategies used to prevent cisplatin-induced nephrotoxicity are actively studied; for example, hydration with magnesium and mannitol supplements [41], the use of various natural and synthetic antioxidants [42], nitric oxide modulators, diuretics, and cytoprotective and anti-apoptotic agents [43,44] such as cilastatin [45,46], vincamine [47], dibenzazepine [48], astaxanthin [49], theophylline [50], and others. It is known that methylene blue has a wide spectrum of action, including cytoprotective, anti-apoptotic, and antioxidant actions [51].

In vitro experiments showed that methylene blue does not lead to a decrease in the level of diene conjugates (Figure 2A) and mtDNA damage (Figure 3) in the kidneys, which are caused by cisplatin exposure. Moreover, methylene blue promotes an increase in H_2_O_2_ production in the intact kidney’s mitochondria (Figure 1), which is consistent with the data obtained earlier on brain mitochondria [52,53,54,55,56]. Presumably, an increase in the rate of H_2_O_2_ production at picomolar concentrations is not capable of causing strong damage of membranes or mtDNA, but H_2_O_2_ can act as a signaling molecule capable of activating some transcription factors, such as nuclear factor erythroid 2-related factor 2 (Nrf2) [57,58]. H_2_O_2_ leads to the oxidation of cysteine residues in kelch-like ECH-associated protein 1 (KEAP1), changing its conformation and inhibiting binding to Nrf2. This process prevents ubiquitination and degradation of Nrf2 [59]. The Nrf2/ARE signaling pathway is considered one of the most important defense mechanisms against oxidative stress [60,61].

It has been demonstrated previously that methylene blue is able to activate the Nrf2/ARE signaling pathway [22,62,63]. In turn, Nrf2-null mice treated with cisplatin showed more pronounced damage of renal cells [64]. We have shown that methylene blue increases the expression of a number of antioxidant genes *Cat*, *Gclc*, *Txnrd2*, *Gpx* (Figure 5), which is apparently associated with the activation of the Nrf2/ARE signaling pathway. Another consequence of the activation of the Nrf2/ARE signaling pathway is the activation of DNA repair pathways. It is known that the main mtDNA repair pathway is the base excision repair (BER) pathway [65]. BER is a DNA repair system that removes damaged bases from the double helix. BER begins with recognition and removal of the damaged base by DNA glycosylases, one of which is 8-Oxoguanine DNA glycosylase (*Ogg1*) [66]. *Brca1* stimulates the activity of key BER enzymes, including *Ogg1* [67]. In turn, Nrf2 can regulate the expression of genes involved in DNA repair, including *Ogg1* [68] and *Brca1* [69]. We have shown that methylene blue increased the expression of *Nfe2l2*, *Ogg1*, and *Brca1* genes (Figure 5). We can hypothesize that activation of the Nrf2/ARE signaling pathway by methylene blue is responsible for the increase in repair activity, resulting in mtDNA protection (Figure 6). Nrf2 also regulates the expression of *Trp53bp1*, a critical intermediate of non-homologous end joining (NHEJ) repair [70]. However, we did not find that methylene blue increased *Trp53bp1* expression, although cisplatin caused a decrease in its expression (Figure 5). This study may indirectly indicate that methylene blue impacts on the BER pathway, not NHEJ pathway.

Azure B is a main metabolite of methylene blue, which forms as a result of the oxidative demethylation of methylene blue. Azure B also has different biological properties and, therefore, can contribute to the pharmacological profile of the compound [71,72]. Methylene blue is structurally similar to azure B, but they differ in the degree of ionization of their oxidized forms. Azure B can deprotonate to some extent with the formation of neutral quinone imine species, which provide its best diffusion through membranes [73]. This is likely why azure B proves to be a safer, and in some cases even more effective, drug than methylene blue [71,72,73,74]. However, we have demonstrated that azure B leads to a smaller increase in the expression of antioxidant and repair genes, which were inhibited by cisplatin (Figure 5). Azure B also prevents mtDNA damage less compared to methylene blue (Figure 4), and does not lead to a decrease in lipid peroxidation products (Figure 2A,B). Therefore, azure B has less pronounced protective properties and is less effective in cisplatin nephrotoxicity compared to methylene blue.

## 4. Materials and Methods

### 4.1. Laboratory Animals

C57BL/6 strain mice (Mus musculus) were used in the experiment. Mice were obtained from the Stolbovaya nursery. Animals were kept under standard conditions: controlled temperature (22–25 °C) and humidity (at least 40%), maintained in a 12-h light–dark cycle, with access to water and food ad libitum. The keeping, injections, and sacrifice of animals were carried out in accordance with the rules established by the Institutional Committee for the Care and Use of Animals of the Voronezh State University (Section of Animal Care and Use, protocol on Biomedical Research 42-03 dated 8 October 2020).

### 4.2. Designs of Experiment

We referred to previous studies when planning experiments. The search for literary sources was carried out in the PubMed database; we analyzed 77 literary sources from the years of 1984 to 2022.

The study included two experiments: in vitro and in vivo. In an in vitro experiment, preliminary isolation of mitochondria from the kidneys was carried out. Further, mitochondria were divided into four aliquots (each tube contains 0.05 mg of mitochondrial protein). The first aliquot (control) was not incubated with any compound; in the second aliquot (cisplatin), mitochondria were incubated with 0.05 mg of cisplatin (Teva Pharmaceutical Industries Ltd., Petah Tikva, Israel) for 30 min. The third (cisplatin + methylene blue) and fourth (cisplatin + azure B) aliquots were pre-incubated for 10 min with 1 μM of methylene blue (Sigma-Aldrich, St. Louis, MO, USA) and 1 μM of azure B (Sigma–Aldrich, St. Louis, MO, USA), respectively, then for 30 min along with 0.05 mg of cisplatin. Incubation was carried out in a shaker Orbital Shaker-Incubator ES-20 (BioSan, Riga, Latvia) at 150 rpm and 37 °C. Then, DNA was isolated from mitochondria for subsequent measurement of the amount of mtDNA damage and the concentration of diene conjugates. Earlier studies have shown that the optimal cisplatin concentration ranges from 0.2–200 µg [75]. We have experimentally established that cisplatin causes mtDNA damage in concentrations from 50 µg.

The in vivo experiment involved 32 mice of both sexes, which were randomly divided into four groups. The first group (*n* = 10) was a control group and received saline injections and pure water, the second group (*n* = 6) was exposed to cisplatin by intraperitoneal injections at a dose of 2 mg/kg/day and also received pure water, the third group (*n* = 8) received injections of cisplatin (2 mg/kg/day) and oral methylene blue at a dosage of 15 mg/kg/day, and the fourth group (*n* = 8) received injections of cisplatin (2 mg/kg/day) and azure B at a dosage of 15 mg/kg/day. Mice received thiazine dye solutions for three weeks. Cisplatin injections were administered daily during the last week of the experiment. Subsequently, the animals were sacrificed, and the kidneys were removed for further molecular and biochemical studies. We have previously shown that cisplatin caused serious cognitive impairment at a concentration of 2 mg/kg/day, and it also caused mice mortality at higher concentrations. For this reason, in this study we settled on a concentration of 2 mg/kg/day [22]. It has previously been shown that methelene blue restores the mitochondrial metabolism of mice at a concentration of 15 mg/kg/day, but not at a concentration of 5 mg/kg/day [53].

### 4.3. Isolation of Mitochondria from the Kidneys

Mice were sacrificed by dislocation of the cervical spine followed by decapitation. The kidneys were quickly removed and placed in an isolation buffer consisting of 225 mM mannitol (Sigma–Aldrich, St. Louis, MO, USA), 75 mM sucrose (Dia–M, Moscow, Russia), 5 mM Hepes (BioClot, Aidenbach, Germany) (pH = 7.4), and 1 mM ethyleneglycoltetraacetic acid (EGTA) (Sigma–Aldrich, St. Louis, MO, USA), and supplemented with 2 mg/mL fatty acid-free bovine serum albumin (BSA) (Dia–M, Moscow, Russia). The wash buffer used in the centrifugation step had the same composition, except for the addition of BSA.

The kidneys were homogenized using a KIMBLE Dounce tissue grinder (Sigma–Aldrich, St. Louis, MO, USA). The resulting homogenate was centrifuged using a Z36 HK centrifuge (Hermle Labortechnik, Wehingen, Germany) for 5 min at 900× *g*. The supernatant was transferred into clean tubes and centrifuged for 10 min at 9000× *g*. Afterwards, the supernatant was removed, and the pellet was resuspended in the wash buffer and centrifuged for 10 min at 9000× *g*. The resulting mitochondrial pellet was resuspended in the wash buffer.

### 4.4. Assessment of the Rate of H_2_O_2_ Production in Mitochondria

H_2_O_2_ production in mitochondria was measured using the Amplex Ultra Red fluorescent reagent (Invitrogen, Carlsbad, CA, USA) according to the protocol described earlier [76]. The Amplex Red assay is a most specific and sensitive method, with a limit of detection less than 5 pmol of H_2_O_2_. The stoichiometry of Amplex Red and H_2_O_2_ is 1:1, and, therefore, the assay results are linear over the range of values encountered in tissues and cells [76]. The excitation wavelength was set to 530 nm and the emission wavelength to 590 nm. The measurements were carried out using a Hitachi F-7000 fluorescence spectrophotometer (Hitachi High Technologies, Tokyo, Japan).

The substrate (10 mM pyruvate), 4 mM phosphate (KH2PO4), 1 U of Amplex Ultra Red reagent, 4 U of horseradish peroxidase (Amresco, Solon, OH, USA), and 0.2 mg of mitochondria were added to 1 mL of isolation buffer (225 mM mannitol, 75 mM sucrose, 5 mM Hepes (pH 7.4), 1 mM EGTA, 2 mg/mL fatty acids free BSA. The H_2_O_2_ concentration was measured as the fluorescence intensity of resorufin formed during the reaction upon oxidation of Amplex Ultra Red. Production changes were recorded after the addition of 0.2 mg cisplatin, 1 μM methylene blue, and 1 μM azure B.

### 4.5. Measurement of Lipid Peroxidation Products

The diene conjugates (DC) are the primary products of lipid peroxidation, and the concentration of DC can be considered a more reliable indicator of the concentration of lipid peroxidation products than measurement of concentration of secondary product of lipid peroxidation [38]. DC concentration was measured by spectrophotometry using a Hitachi U-2900 spectrophotometer (Hitachi High-Technologies, Tokyo, Japan). In the in vivo experiment, the frozen kidney was preliminarily weighed and homogenized in 0.5 mL of PBS. Normalization of DC concentration was performed relative to the mass of the kidney. In the in vivo experiment, the preliminary isolation of mitochondria was carried out. Normalization of DC concentration was performed relative to the protein concentration.

0.125 mL of saline (MOSPHARM, Moscow, Russia), 1.5 mL of heptane, and 1.5 mL of isopropyl alcohol (RFK, Moscow, Russia) were added to 125 µL of probe. The resulting mixture was centrifuged for 10 min at 3000× *g* and 4 °C. Then, distilled water was added to the supernatant in a ratio of 10:1, and the phases were expected to separate. The upper heptane phase was transferred into a clean test tube and 0.5 mL of ethyl alcohol was added in a ratio of 1:5, and 96% ethanol served as a control.

Calculation of the DC concentration was performed according to the formula:

[DC] = (Vtot × D × 10^6^)/(L × E × m × Vin)

where, Vtot is the sample volume (0.5 mL); D is the optical density value; L is the length of the optical path (1 cm); E is the coefficient of molar extinction (2.2 × 10^5^); m is the mass of the kidney (in vivo experiment) and the amount of added protein (in vitro experiment); Vin is the volume of the introduced sample (0.125 µL).

### 4.6. Measuring the mtDNA Damage Level

The non-PCR-based methods for evaluating the amount of DNA damage, such as high-performance liquid chromatography and Southern blot, have a number of limitations. In particular, they require considerable amounts of DNA for analysis (10–50 µg). Cells contain a small amount of mtDNA compared to nuclear DNA, so its analysis requires more sensitive methods, such as long-range PCR [77].

The total DNA isolation in the in vivo experiment was performed using the DNA-sorb-S-M kit (AmpliSens, Moscow, Russia) according to the protocol. In an in vitro experiment, the total DNA from mitochondria was isolated using the Proba-GS kit (DNA-Technology, Moscow, Russia).

The level of mtDNA damage was measured by a quantitative real-time PCR using the CFX96 Touch (Bio-Rad, Hercules, CA, USA). The reaction mixture (volume 20 µL) included 4 µL of 5X qPCRmix-HS SYBR (Evrogen, Moscow, Russia), 1 µL mixture of forward and reverse primers, 1 µL DNA, and 14 µL mQ water. Reaction conditions were: total denaturation was carried out at 95 °C for 3 min; denaturation at the beginning of the cycle at 95 °C for 30 s; primer annealing at 59 °C for 30 s, elongation at 72 °C for 4 min 30 s; number of cycles was 38; a melting curve from 65 °C to 95 °C, according to protocol described earlier [77]. In the experiment, the 1st, 2nd, 3rd, 7th, 8th, and 9th long fragments were used, because these fragments did not have nuclear pseudogenes.

To determine the degree of mtDNA damage, the ΔCq value of the control and experimental (damaged) long fragments was compared with the ΔCq value of the control and experimental short fragments, which were used as a reference.

The number of mtDNA lesions was calculated per 10 kb according to the formula:

Lesions = (1 − E^−(∆long−∆short)^) × 10,000 (bp)/fragment length (bp)

where Δ long = Cq control − Cq experiment for the long fragment and Δshort = Cq control − cq experiment for the short fragment.

The primer sequences were as follows (Table 1):

### 4.7. Estimation of Gene Expression Level

Total RNA was isolated using the ExtractRNA kit (Evrogen, Moscow, Russia), according to the protocol. Reverse transcription was performed on a personal Eppendorf Mastercycler (Eppendorf, Hamburg, Germany). RNA at 9 µL and Random primer at 2 µL were mixed and heated in an amplifier at 70 °C for 2 min to anneal the primers. The following components were added to the mixture: 2 µL dNTP, 2 µL mQ water, 1 µL M-MULV revertase, and 4 µL 5X buffer (both Evrogen, Moscow, Russia). The mixture was incubated for 1 h at 35 °C.

The level of gene expression was assessed using quantitative PCR analysis. The reaction mixture (volume 20 µL) included: 4 µL of 5X qPCRmix-HS SYBR (Evrogen, Moscow, Russia), 1 µL mixture of forward and reverse primers, 1 µL DNA, and 14 µL mQ water. Reaction conditions were: total denaturation was carried out at 95 °C for 3 min; denaturation at the beginning of the cycle at 95 °C for 30 s; primer annealing at 59 °C for 30 s, elongation at 72 °C for 30 s; number of cycles was 45; melting curve from 65 °C to 95 °C.

The primer sequences were as follows (Table 2):

### 4.8. Statistical Analysis

Statistical analysis was carried out using the Statistica 10 software package (StatSoft, Tulsa, OK, USA). Results are presented as means ± S.E.M. The statistical significance of differences between groups was assessed using the Mann–Whitney test (U-test). Statistical significance was considered to be *p* < 0.05.

## 5. Conclusions

The cisplatin-induced lesions of mitochondria and mtDNA are one of the main limitations to its widespread use in medicine. Methylene blue, a well-known mitochondrial protector, reduced the amount of mtDNA damage by triggering repair processes, likely through the Nrf2/ARE pathway activation. It is likely that methylene blue or some other Nrf2 activators can serve as drugs that reduce the toxicity of cisplatin to non-tumor tissues, particularly in the kidneys.

## Figures and Tables

**Table 1:** Primers that were used to analyse measurement of mtDNA damage.

Fragment Name	Primers Sequence
2 short	Forward: 5′-ACGAGGGTCCAACTGTCTCTTA-3′ Reserve: 5′-AGCTCCATAGGGTCTTCTCGT-3′
1 long	Forward: 5′-TAAATTTCGTGCCAGCCACC-3′ Reserve: 5′-ATGCTACCTTTGCACGGTCA-3′
2 long	Forward: 5′-ACGAGGGTCCAACTGTCTCTTA-3′ Reserve: 5′-CCGGCTGCGTATTCTACGTT-3′
3 long	Forward: 5′-CTAGCAGAAACAAACCGGGC-3′ Reserve: 5′-TTAGGGCTTTGAAGGCTCGC-3′
7 long	Forward: 5′-TCATTCTTCTACTATCCCCAATCC-3′ Reserve: 5′-TGGTTTGGGAGATTGGTTGATG-3′
8 long	Forward: 5′-CCCCAATCCCTCCTTCCAAC-3′ Reserve: 5′-GGTGGGGAGTAGCTCCTTCTT-3′
9 long	Forward: 5′-AAGAAGGAGCTACTCCCCACC-3′ Reserve: 5′-GTTGACACGTTTTACGCCGA-3′

**Table 2:** Primers that were used to analyze gene expression.

Gene Name	Primers Sequence
*Gapdh*	Forward: 5′-GGCTCCCTAGGCCCCTCCTG-3′; Reserve: 5′-TCCCAACTCGGCCCCCAACA-3′;
*Nfe2l2*	Forward: 5′-CTCTCTGAACTCCTGGACGG-3′ Reserve: 5′-GGGTCTCCGTAAATGGAAG-3′
*Ho1*	Forward: 5′-CACGCATATACCCGCTACCT-3′ Reserve: 5′-CCAGAGTGTTCATTCGAGCA-3′
*p62*	Forward: 5′-GCCAGAGGAACAGATGGAGT-3′ Reserve: 5′-TCCGATTCTGGCATCTGTAG-3′
*Pink1*	Forward: 5′-GAGCAGACTCCCAGTTCTCG-3′ Reserve: 5′-GTCCCACTCCACAAGGATGT-3′
*Prdx3*	Forward: 5′-GTGGTTTGGGCCACATGAAC-3′ Reserve: 5′-TGGCTTGATCGTAGGGGACT-3′
*Gpx*	Forward: 5′-AGTCCACCGTGTATGCCTTCT-3′ Reserve: 5′-GAGACGCGACATTCTCAATGA-3′
*Txnrd2*	Forward: 5′-GATCCGGTGGCCTAGCTTG-3′ Reserve: 5′-TCGGGGAGAAGGTTCCACAT-3′
*Gclc*	Forward: 5′-GGGGTGACGAGGTGGAGTA-3′ Reserve: 5′-GTTGGGGTTTGTCCTCTCCC-3′
*Sod2*	Forward: 5′-CAGACCTGCCTTACGACTATGG-3′ Reserve: 5′-CTCGGTGGCGTTGAGATTGTT-3′
*Cat*	Forward: 5′-AGCGACCAGATGAAGCAGTG-3′ Reserve: 5′-TCCGCTCTCTGTCAAAGTGTG-3′
*Ogg1*	Forward: 5′-GAGACGACAGCCAGGTGTGAG-3′ Reserve: 5′-CCGTTCCACCATGCCAGTA-3′
*Trp53bp1*	Forward: 5′-GAAGGAAAGCACAGATGAGGATT-3′ Reserve: 5′-CTAGAGGTTTCTGCACGCTG-3′
*Brca1*	Forward: 5′-AGGTGATTGCAGTGTGAGAGA-3′ Reserve: 5′-GTATCCGGATGCCTCCTCTTC-3′

## Data Availability

Data are available from the corresponding authors upon request.
